# Health implications of safe drinking water act violations: a county-level analysis

**DOI:** 10.3389/fpubh.2025.1588338

**Published:** 2025-08-05

**Authors:** Fahad Alzahrani, Khalid Alhussain

**Affiliations:** ^1^Department of Agribusiness and Consumer Sciences, King Faisal University, Al-Ahsa, Saudi Arabia; ^2^Department of Pharmacy Practice, King Faisal University, Al-Ahsa, Saudi Arabia

**Keywords:** drinking water contamination, health-based violations, self-reported health outcomes, BRFSS, beta regression

## Abstract

Health-based drinking water violations impact millions of Americans each year. This study examines the relationship between 13 measures of drinking water quality, related to health-based violations of the Safe Drinking Water Act, and three self-reported health outcomes (general, physical, and mental health). Analyzing cross-sectional data from 3,100 counties in the United States using regression analysis, we found statistically significant relationships between health-based violations and all self-reported health outcomes. Specifically, counties with more health-based violations reported a higher percentage of people with fair or poor health, and more physically and mentally unhealthy days. Our findings indicate that a single health-based violation in an average county incurs yearly medical costs of approximately $3.48 million for physical health and $4.85 million for mental health. These results highlight the need for policymakers and health professionals to prioritize interventions that address these violations, particularly in vulnerable communities, to mitigate their long-term health impacts.

## 1 Introduction

Access to safe and clean drinking water is considered a human right (1). However, about 2.2 billion people around the world, including in developed countries, lack access to safely managed drinking water (2). This statistic highlights a wider issue that also affects communities in the United States (U.S.). A recent Gallup poll (2019–2023) found that drinking water contamination is the main environmental concern among U.S. citizens (3). More than 90% of Americans rely on public water systems (PWSs) as their primary source of drinking water, yet the level of service provided to water customers varies across the country (4, 5). Factors such as aging infrastructure, limited financial and human resources, and impaired water sources contribute to these disparities. Recent studies have shown that many PWSs struggle to provide safe drinking water to millions of customers in many parts of the U.S. (6–8). This can result in negative health consequences. For example, between 2015 and 2020, health departments in 28 states reported 214 outbreaks associated with drinking water and 454 contributing factor types (9). Poor drinking water quality therefore can result in significant health costs (10). Recent estimates by (11) indicated that each year, waterborne pathogens cause about 7.2 million illnesses, 120,000 hospitalizations, and 6,660 deaths, resulting in $3.3 billion in direct healthcare costs.

The relationship between drinking water quality and health has been well-established since the mid-1850s when John Snow linked cholera epidemics to contaminated water in London (12). Many studies have investigated the impact of drinking water quality on health outcomes in the U.S. These studies can be categorized into two types. The first type includes research that relied on primary data, where researchers collected on-site samples, tested them for contaminants, and examined the effects on health outcomes (13, 14). The second category includes studies that utilized secondary data from Safe Drinking Water Information System (SDWIS) to assess drinking water quality. In these studies, drinking water quality was measured in various ways, including the concentrations of specific contaminants such as arsenic or nitrate at the community water system (CWS), city, or county levels. Other measures also include whether the CWS had a health-based violation; number of health-based violations; and percentage of population exposed to contaminations. In this paper, we focused on the second category, as we followed the same methodology in measuring drinking water quality.

The majority of the studies using SDWIS focused on the impact of drinking water quality on birth and pregnancy-related health outcomes (15–29). The rest examined the effect of drinking water quality on the risk of different types of cancer or emergency department visits for gastrointestinal illness (30–33). One study examined the relationship between increased chromium concentrations in drinking water, which works as an antidepressant, and suicide rates in Alabama (34).

To our knowledge, no study has examined the impact of drinking water quality on health outcomes related to quality of life such as physical and mental health measures. Physical and mental health measures are essential domains of quality of life assessment; including such outcome measures in research helps capture the full impact of drinking water quality on individuals. Moreover, most of the previous studies limited their analysis to one state. Therefore, the aim of this study is to contribute to the growing body of evidence that underscores the necessity of ensuring clean drinking water by examining the impact of Safe Drinking Water Act (SDWA) health-based violations on three health outcome measures including general, physical, and mental health at the county level in the contiguous U.S.

## 2 Materials and methods

### 2.1 Data sources

We relied on three sources of data. The first was the Behavioral Risk Factor Surveillance System (BRFSS) survey for health outcome variables. The BRFSS is a national program for collecting state-specific data through health-related telephone surveys. It gathers information on U.S. residents’ health behaviors, chronic conditions, access to healthcare, and use of preventive services. Established in 1984, the BRFSS operates in all 50 states, the District of Columbia, and U.S. territories, conducting over 400,000 adult interviews annually, making it the largest and longest-running telephone health survey in the world (35). The self-reported health measures from the BRFSS survey have been widely used in the health literature to study the relationship between health and environmental factors (36–39). The data are accessible at the county level by the County Health Rankings & Roadmaps, developed by the Robert Wood Johnson Foundation and the University of Wisconsin Population Health Institute.

The second source of data was the Environmental Protection Agency’s (EPA) Safe Drinking Water Act Information System (SDWIS) for drinking water quality variables. The SDWIS was developed in 1995, but it did not become publicly available until 2013 (40, 41). The Safe Drinking Water Act (SDWA) establishes Maximum Contaminant Levels (MCLs) for various contaminants permissible in drinking water without adversely affecting human health. All water systems in the U.S. must comply with these standards. Water systems regularly test their water and exceeding these limits results in a violation of the SDWA. The EPA categorizes violations into three tiers based on the immediacy of public health risk. Tier 1 violations are the most serious and pose an immediate health risk, such as the presence of fecal coliform in a water system sample. Tier 2 violations can lead to severe health effects, typically after prolonged exposure; arsenic is one example of a contaminant in this category. Tier 3 violations are related to monitoring and reporting requirements and do not pose a health risk. Since we were interested in examining the health impacts of poor drinking water quality, we used the data for Tier 1 and Tier 2 violations.

The third source was the U.S. Census American Community Survey (ACS). We used the 5-year estimates from the ACS to obtain socioeconomic and demographic variables for each county. Our analysis was based on cross-sectional data collected from 3,100 counties in the contiguous U.S. for the year 2021. The three datasets were linked using the Federal Information Processing Standards (FIPS) codes, which were available across all three datasets.

### 2.2 Measures

#### 2.2.1 Dependent variables: self-reported health outcomes

In this study, three self-reported health outcome variables were used as dependent variables. The first variable was the percentage of adults reporting fair or poor general health, based on responses to the BRFSS survey question: “Would you say that in general your health is Excellent/Very good/Good/Fair/Poor?” The second measure was the average number of physically unhealthy days reported per month, calculated from the question: “Now thinking about your physical health, which includes physical illness and injury, for how many days during the past 30 days was your physical health not good?” The final health measure was the average number of mentally unhealthy days reported per month, based on responses to the question: “Now thinking about your mental health, which includes stress, depression, and problems with emotions, for how many days during the past 30 days was your mental health not good?”

#### 2.2.2 Key independent variables: drinking water quality

A total of 13 variables related to drinking water quality were included in the analysis (see Table 1). To measure drinking water quality at the county level, we used health-based drinking water violations from SDWIS. All health-based violations from active community water systems were included in the analysis. The dataset contains various information about these violations, such as water system name, ID, other characteristics (water source and population served), type of violation, and when the violation started and ended. The dataset also provides information about the location of the water system and principal county served. Following the methodology used by Allaire et al. (42), we created multiple variables related to drinking water violations at the county level, including whether the county had a violation, the total number of Tier 1 and Tier 2 violations (i.e., sum of all violations from all CWSs serving that county), and the percentage of population affected by these violations. Additionally, we created variables for violations related to pathogens, a category of Tier 1 violations, which includes those caused by excessive turbidity levels or the confirmed presence of fecal coliform or *E. coli.* Finally, we calculated the duration of each violation category based on the start and end dates of the compliance period.

**Table 1:** Summary statistics (observations = 3,100 counties).

Variable	Mean	Std. Dev.	Min	Max	Description
Health variables					
Poor general health	17.7454	4.5298	8.4000	38	Percentage of adults reporting fair or poor health
Poor physical	3.8980	0.6433	2.2618	6.4495	Average number of physically unhealthy days reported per month
Poor mental	5.2213	0.6115	3.1855	7.3888	Average number of mentally unhealthy days reported per month
Drinking water quality variables					
Violation event	0.3432	0.4749	0	1	1 = County is affected by drinking water violation, 0 = Otherwise
Violations	2.0761	6.9509	0	117	Total number of health-based violations
Pop affected by violations	4.8015	14.1604	0	100	Percentage of population affected by health-based violations
Violations duration	38.8117	84.5997	0	858	Average duration in days of all health-based violations
T1 violations	0.2619	1.5537	0	36	Total number of tier 1 health-based violations
Pop affected by T1 violations	1.4980	8.7641	0	100	Percentage of population affected by tier 1 violations
T1 violations duration	5.4610	25.4835	0	455	Average duration in days of tier 1 health-based violations
T1 violations (pathogens)	0.1081	0.7491	0	20	Total number of tier 1 health-based violations related pathogens
Pop affected by T1 violations (pathogens)	1.1093	7.7003	0	100	Percentage of population affected by tier 1 pathogens
T1 violations (pathogens) duration	3.6347	18.5158	0	333	Average duration in days of tier 1 health-based violations related to pathogens
T2 violations	1.8142	6.3991	0	110	Total number of tier 2 health-based violations
Pop affected by T2 violations	4.3107	13.5434	0	100	Percentage of population affected by tier 2 violations
T2 violations duration	36.9403	85.7055	0	858	Average duration in days of tier 2 health-based violations
Socioeconomic and demographic variables					
Education	22.9663	9.9075	0	78.7	Percentage of population 25 years and over with bachelor’s degree or higher
Income	30.4532	7.5337	10.3010	83.0080	Per capita income (in $000 Inflation-Adjusted Dollars)
Poverty	14.4460	6.1169	1.2	59	Percentage of population below poverty level
Unemployment	5.1986	2.5888	0	32.4	Percentage of unemployed in the civilian labor force (unemployment rate)
Uninsured	9.6299	5.0770	0	44.9	Percentage of population without health insurance
Gini	44.56334	3.7809	24.93	72.7900	Gini index (measures income inequality, 0 = perfect equality and 100 = perfect inequality)
Older adult	19.2506	4.7036	3.0735	57.6260	Percentage of the population who are 65 years old and over
Nonwhite	19.2023	16.5979	0	95.6849	Percentage of Nonwhite population

#### 2.2.3 Other independent variables: socioeconomic and demographic variables

The variables in this category included education, poverty, income, unemployment, the Gini coefficient, older adult population, and nonwhite population. Since data were not available for some areas, specifically Connecticut, we excluded them from the regression analysis.

### 2.3 Statistical analysis

We used summary statistics to describe and visualize the data. We also used regression analysis to examine the relationships between drinking water quality and health outcomes. We modeled this relationship using the following specification:

(1) $$y_{i}=\beta_{0}+\beta_{1}x_{1}+\beta_{2}x_{2}+\ldots +\beta_{k}x_{k}+\varepsilon_{i}$$

where $y_{i}$ is the health outcome measures for county i , $\beta_{0}$ is the intercept, $x_{1}$ measures one violation variable, such as total number of violations or the percentage of population affected by violations in county i , $x_{2}$ and $x_{k}$ represent the control variables (socioeconomic and demographic variables), and $\varepsilon_{i}$ is the error term.

We estimated the above equation using Ordinary Least Squares (OLS) to obtain the estimates for the parameters β . However, because one of our health outcome variables is a percentage (poor general health), using the linear regression model in Equation 1 for this variable is not appropriate, as it does not guarantee that the predicted values of the dependent variable are restricted to the unit interval (43). In such cases, fractional regression models, such as logit transformation and beta regression, are used to study the relationship between bounded dependent variables and independent variables. In our analysis, we employed the beta regression model proposed by Ferrari and Cribari-Neto (44). We selected this model because it overcomes key limitations of the logit transformation model (45). This model assumes that the response variable has a beta distribution, meaning the data must be expressed as rates or proportions. Specifically, the response variable must be 0<y<1 . This fits our data, as shown in Table 1, where poor general health is between 0.084 and 0.380. The beta regression typically uses a logit link function to relate the independent variables to the mean of the response variable.

## 3 Results

### 3.1 Descriptive statistics

**Figure 1:**
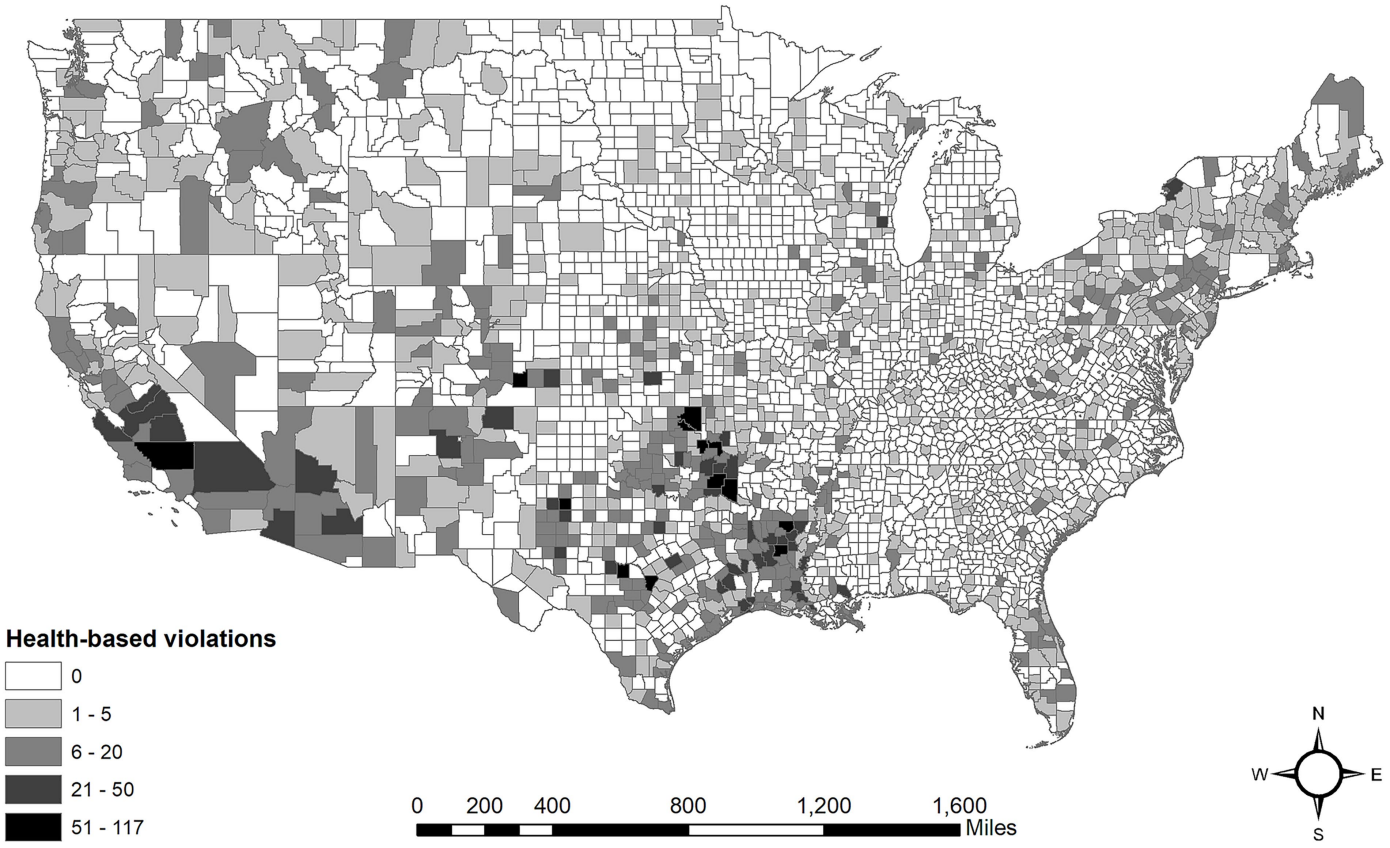
Total number of health-based drinking water violations 2021.

Table 1 provides summary statistics and descriptions for all variables included in the analysis. We found that about third of the counties in the U.S. experienced at least one health-based violation in 2021. The average number of health-based violations was 2.08, with a standard deviation of 6.95, reflecting substantial variability, as the total number of violations ranged from 0 to 117. Furthermore, about 4.80% of the population in affected counties faced health-based violations, with a standard deviation of 14.16, indicating that some counties had a much larger proportion of their population affected. Lastly, the average duration of these health-based violations was 38.81 days, with a standard deviation of 84.60, suggesting that some violations can persist for extended periods, ranging from 0 to 858 days. Figure 1 shows the distribution of health-based violations across the counties. The distribution shows significant clustering, with particularly high violation densities in rural counties of the Southern U.S. (Texas, Louisiana, and Oklahoma), Southwest, and Northeast regions. These areas tend to include CWSs with repeat violations. In our sample, about 13% of the CWSs have repeat violations (i.e., five or more health-based violations).

Upon examining health-based violations more closely, it is evident that Tier 1 violations were less prevalent and shorter in duration compared to Tier 2 violations. The average number of Tier 1 violations was 0.26, while Tier 2 violations had a higher average of 1.81. Additionally, Tier 1 violations last an average of 5.46 days, significantly shorter than the 36.94 days for Tier 2. Furthermore, a smaller percentage of the population was affected by Tier 1 violations, impacting 1.50% compared to 4.31% for Tier 2 violations. In terms of violations related to pathogens, the average was 0.11 and affected 1.11% of the population, with an average duration of 3.63 days.

For self-reported health outcome variables, on average, 17.75% of adults in U.S. counties reported fair or poor health, with a standard deviation of 4.53, indicating moderate variability across different counties, where values ranged from 8.40 to 38%. In terms of physical health, individuals reported an average of 3.90 physically unhealthy days per month, with a standard deviation of 0.64, suggesting that most counties experience a similar number of unhealthy days, ranging from 2.26 to 6.45 days. Additionally, the average number of mentally unhealthy days was 5.22, with a standard deviation of 0.61, reflecting relatively low variability, as reported days ranged from 3.19 to 7.39.

### 3.2 Regression results

Table 2 summarizes the results from the regression models analyzing the relationship between drinking water quality variables and self-reported health outcomes. In total, we estimated 39 regression models (13 drinking water quality variables × 3 health outcome variables). Each regression model included the socioeconomic and demographic covariates (full models are available in the Supplementary material).

**Table 2:** Regression results for drinking water quality variables.

Dependent variable	Poor general health (1)	Poor general health (Marginal effects) (2)	Poor physical (3)	Poor mental (4)
Violation event	0.0143*** (0.0046)	0.0007*** (0.0002)	0.0246** (0.0122)	0.0605*** (0.0170)
Number of violations	0.0012*** (0.0002)	0.0004*** (0.0001)	0.0020*** (0.0006)	0.0044*** (0.0010)
Population affected by violations	0.0894*** (0.0132)	0.0007*** (0.0001)	0.0016*** (0.0004)	0.0025*** (0.0005)
Violations duration	0.0000 (0.0000)	0.0001 (0.0001)	0.0000 (0.0001)	0.0002* (0.0001)
T1 violations	0.0033*** (0.0009)	0.0001*** (0.0000)	−0.0012 (0.0026)	−0.0023 (0.0049)
Pop affected by T1 violations	0.0714*** (0.0157)	0.0002*** (0.0000)	0.0000 (0.0005)	0.0008 (0.0007)
T1 violations duration	0.0001* (0.0001)	0.0001* (0.0001)	−0.0003 (0.0002)	−0.0002 (0.0003)
T1 violations (pathogens)	0.0030* (0.0018)	0.0001* (0.0000)	0.0069 (0.0062)	0.0275** (0.0112)
Population affected by T1 violations (pathogens)	0.0703*** (0.0172)	0.0001*** (0.0000)	0.0004 (0.0006)	0.0012 (0.0008)
T1 violations (pathogens) duration	0.0001 (0.0001)	0.0000 (0.0001)	0.0001 (0.0002)	0.0005 (0.0004)
T2 violations	0.0012*** (0.0002)	0.0003*** (0.0001)	0.0025*** (0.0007)	0.0054*** (0.0011)
Population affected by T2 violations	0.0897*** (0.0137)	0.0006*** (0.0001)	0.0017*** (0.0004)	0.0027*** (0.0005)
T2 violations duration	0.0000 (0.0000)	0.0001 (0.0001)	0.0001 (0.0001)	0.0002** (0.0001)

Robust standard errors in parentheses; *** *p* < 0.01, ** *p* < 0.05, * *p* < 0.1. Each estimate represents a separate model with all controls included using 3,100 observations. The results in column (1) present the coefficients obtained from beta regression models, which estimate the impact of drinking water quality variables on the percentage of adults reporting fair or poor health. Column (2) provides the average marginal effect to facilitate the interpretation of these results. Columns (3) and (4) display the OLS results for the impact of drinking water quality variables on the average number of poor physically and mentally unhealthy days reported per month.

The results indicate that violation events have a positive and statistically significant impact on health outcomes. Specifically, when a violation event occurs in a county, the percentage of adults reporting fair or poor health (Poor general health) increases by 0.07%. This effect is also notable for the average number of poor physically (Poor physical health) and mentally (Poor mental health) unhealthy days reported per month, showing increases of 0.03 and 0.06 days, respectively. Additionally, the total number of violations is associated with negative health impacts across all outcomes. Each additional violation corresponds to an approximate increase of 0.04% in poor general health, and increases of 0.002, and 0.004 days in poor physical health and poor mental health, respectively.

When considering the percentage of the county population affected by violations, we found that a 10% increase in this population resulted in increases of 0.7%, 0.016 days, and 0.025 days in poor general health, poor physical health, and poor mental health, respectively. While the duration of violations did not show statistically significant impacts on two health outcomes, it did have a minor but statistically significant effect on poor mental health, indicating an increase in the average number of mentally unhealthy days reported per month.

Regarding the total number of Tier 1 violations, we observed a small but statistically significant effect on poor general health, where one additional Tier 1 violation resulted in an increase of 0.01% in the percentage of adults reporting poor general health. Moreover, the percentage of population affected by Tier 1 violations has a statistically significant impact on poor general health; specifically, a 10% increase in this population leads to a 0.2% increase in poor general health. We found similar results for Tier 1 violations related to pathogens concerning poor general health. However, we also found a statistically significant impact on poor mental health, where one additional Tier 1 violation related to pathogens results in an increase of 0.03 days in the average number of mentally unhealthy days reported per month.

For Tier 2 violations, the results revealed statistically significant relationships across all health outcomes, both for the total number of Tier 2 violations and the percentage of population affected by these violations. For example, one additional Tier 2 violation results in increases of 0.06%, 0.002 days, and 0.003 days in poor general health, poor physical health, and poor mental health, respectively. Additionally, the duration of Tier 2 violations has statistically significant impact on poor mental health; for each additional day of Tier 2 violations, there is an increase of 0.0002 days in the average number of mentally unhealthy days reported per month.

## 4 Discussion

The study findings revealed significant associations between health-based violations and self-reported health outcomes across various dimensions. These findings are in line with prior research that links water quality to population health (18, 19, 30).

We found that the occurrence of at least one health-based violation in a county was linked to a 0.07% increase in the proportion of adults reporting fair or poor health and modest increases in the number of physically and mentally unhealthy days reported each month. Although the effect of these violations may appear small, it has meaningful implications for public health, especially when considered across larger populations or over extended periods. For instance, if health-based violations are frequent or widespread across counties, these minor increases could add up and lead to a significant public health burden. In addition, chronic and frequent exposure to such violations can contribute to an overall decline in health-related quality of life and increased healthcare costs (10). For example, using the cost estimates for unhealthy days provided by McNamara et al. (46), our results indicate that for an average county, the yearly medical costs associated with the occurrence of a single health-based violation are $3.48 million and $4.85 million for physically and mentally unhealthy days, respectively. However, with each additional violation, the effect will become less pronounced (see Supplementary Tables 4–6 for more details). These estimates are to be interpreted with caution since McNamara et al. (46) used a cross-sectional design in a specific region in the U.S., which may limit the generalizability of our cost estimates of drinking water violations.

To better understand the associations of health-based violations with self-reported health outcomes, we examined the influence of the total number of violations. Our results indicated that each additional violation corresponds to a small but notable increase in adverse health outcomes. This indicates the cumulative nature of violations and their potential to create significant public health burdens over time. Thus, counties with a high number of violations should be targeted for improvement. Moreover, the proportion of the population affected by violations has been shown to significantly affect the three self-reported health outcomes. For example, a 10% increase in the affected population resulted in noticeable increases in poor general health and both physical and mental health deterioration, suggesting the broader societal implications of widespread environmental violations.

When investigating the types of health-based violations, we observed differences between the effects of Tier 1 and Tier 2 violations. Tier 1 violations had a small but significant effect on poor general health and poor mental health outcomes. This could be due to the fact that Tier 1 violations are more often related to pathogens, and previous studies have established negative relationships between pathogenic organisms and mental health (47, 48). These effects may occur through multiple pathways, including physiological impacts on brain function and psychological stress related to illness or the fear of contamination. Prior research has demonstrated that disruptions to the gut microbiota can influence brain function, highlighting one possible biological mechanism (49). Psychologically, the anxiety or fear of contracting a waterborne illness may also contribute to or exacerbate existing mental health conditions (50). On the other hand, Tier 2 violations had a substantial effect on all three self-reported health outcomes. For example, each additional Tier 2 violation increased the percentage of adults reporting fair or poor general health by 0.06% and contributed to slight increases in poor physical and mental health. This can be explained by the fact that unlike Tier 1 violations, Tier 2 violations typically involve chronic chemical contaminants like arsenic, lead or disinfection byproducts that are known to disrupt multiple physiological systems over time. For example, long-term exposure to arsenic from drinking water and food can cause cancer and skin lesions (51). It has also been associated with cardiovascular disease and diabetes. In utero and early childhood exposure has been linked to negative impacts on cognitive development and increased deaths in young adults. This supports the understanding that more severe and persistent violations tend to have a stronger impact on public health.

Given the above-mentioned considerations, health-based violations in the U.S. should be addressed proactively. Even small violations can have wide-reaching consequences for public health, particularly if they persist over time or are prevalent across many areas. Policymakers and health professionals should consider the long-term implications of these violations and prioritize interventions to mitigate their effects, ensuring healthier communities and reducing the overall impact on public health. For example, stricter enforcement of environmental regulations, especially in counties with a high number of health-based violations, can help to enhance compliance and ensure a consistent supply of safe drinking water. Moreover, improving community-based health services and increasing access to healthcare for those affected by health violations could help mitigate the broader public health impact.

Our study has several strengths and limitations. A major strength was that the study used multiple nationwide, detailed, county-level data, which allowed for a comprehensive analysis. In our analysis, we examined the effects of 13 health-based violation variables on three different health outcomes. Furthermore, we examined a broad range of health outcomes from a population-level perspective and accounted for key socioeconomic and demographic factors. However, we did not control for variables such as access to healthcare, local environmental policies, or other community-specific stressors. Another limitation of our study is its cross-sectional design, which inherently limits the ability to draw causal inferences. In this design, both the independent variables (i.e., health-based violations) and the dependent variables (i.e., self-reported health outcomes) were measured simultaneously, making it difficult to establish a clear temporal relationship between exposure and outcome. For instance, some violations may have been recorded after the BRFSS health data were collected, introducing the potential for temporal misclassification. Conversely, areas with poorer health outcomes may have been subject to increased violations. Such ambiguities in timing can introduce bias and confounding, highlighting the need for longitudinal studies to better assess causal pathways. Additionally, the use of self-reported health outcome variables introduces potential sources of bias, including recall and social desirability bias. Individuals may inaccurately recall their health status or deliberately misreport it to align with perceived social norms, particularly in communities where certain conditions (e.g., mental illness) are stigmatized. Prior research has shown that respondents may underreport poor health behaviors or outcomes due to social pressures or fear of judgment (52). Such biases can lead to systematic misclassification, potentially distorting the observed associations between environmental exposures and health outcomes. Overall, our findings highlight the significant role that health-based violations play in shaping public health outcomes. Future research should continue to explore the long-term effects of such violations and strategies for mitigating their impact, particularly in vulnerable populations.

## 5 Conclusion

By examining the relationship between drinking water quality and self-reported health outcomes at the county level, this study found that health-based violations of the Safe Drinking Water Act (SDWA) do indeed have statistically significant negative effects on general, physical, and mental health. Although the estimated effects are small, the cumulative effect can have large consequences on health outcomes and the associated medical costs. Therefore, policymakers and health professionals should consider the long-term implications of these violations and prioritize interventions to mitigate their effects, especially in vulnerable counties.

## Data Availability

The original contributions presented in the study are included in the article/Supplementary material, further inquiries can be directed to the corresponding author.
