# Supplementary material for: Health implications of safe drinking water act violations: a county-level analysis
Source: Front Public Health. 2025 Aug 5;13:1588338. doi: 10.3389/fpubh.2025.1588338 (PMC12361233; doi:10.3389/fpubh.2025.1588338)
Supplement: Supplementary file 1 [file Data_Sheet_1.docx]

Supplementary Material

# Supplementary Tables

Table 1. Beta regression results – Dependent variable: Poor general health

|  | (1) | (2) | (3) | (4) | (5) | (6) | (7) | (8) | (9) | (10) | (11) | (12) | (13) |
| --- | --- | --- | --- | --- | --- | --- | --- | --- | --- | --- | --- | --- | --- |
| Violation event | 0.014*** |  |  |  |  |  |  |  |  |  |  |  |  |
|  | (0.005) |  |  |  |  |  |  |  |  |  |  |  |  |
| Violations |  | 0.001*** |  |  |  |  |  |  |  |  |  |  |  |
|  |  | (0.000) |  |  |  |  |  |  |  |  |  |  |  |
| Pop affected by violations |  |  | 0.089*** |  |  |  |  |  |  |  |  |  |  |
|  |  |  | (0.013) |  |  |  |  |  |  |  |  |  |  |
| Violations duration |  |  |  | 0.000 |  |  |  |  |  |  |  |  |  |
|  |  |  |  | (0.000) |  |  |  |  |  |  |  |  |  |
| T1 violations |  |  |  |  | 0.003*** |  |  |  |  |  |  |  |  |
|  |  |  |  |  | (0.001) |  |  |  |  |  |  |  |  |
| Pop affected by T1 violations |  |  |  |  |  | 0.071*** |  |  |  |  |  |  |  |
|  |  |  |  |  |  | (0.016) |  |  |  |  |  |  |  |
| T1 violations duration |  |  |  |  |  |  | 0.000* |  |  |  |  |  |  |
|  |  |  |  |  |  |  | (0.000) |  |  |  |  |  |  |
| T1 violations (pathogens) |  |  |  |  |  |  |  | 0.003* |  |  |  |  |  |
|  |  |  |  |  |  |  |  | (0.002) |  |  |  |  |  |
| Pop affected by T1 violations (pathogens) |  |  |  |  |  |  |  |  | 0.070*** |  |  |  |  |
|  |  |  |  |  |  |  |  |  | (0.017) |  |  |  |  |
| Pathogens violations duration |  |  |  |  |  |  |  |  |  | 0.000 |  |  |  |
|  |  |  |  |  |  |  |  |  |  | (0.000) |  |  |  |
| T2 violations |  |  |  |  |  |  |  |  |  |  | 0.001*** |  |  |
|  |  |  |  |  |  |  |  |  |  |  | (0.000) |  |  |
| Pop affected by T2 violations |  |  |  |  |  |  |  |  |  |  |  | 0.090*** |  |
|  |  |  |  |  |  |  |  |  |  |  |  | (0.014) |  |
| T2 violations duration |  |  |  |  |  |  |  |  |  |  |  |  | 0.000 |
|  |  |  |  |  |  |  |  |  |  |  |  |  | (0.000) |
| Education | -1.189*** | -1.183*** | -1.180*** | -1.183*** | -1.183*** | -1.185*** | -1.184*** | -1.184*** | -1.184*** | -1.183*** | -1.183*** | -1.177*** | -1.183*** |
|  | (0.041) | (0.041) | (0.041) | (0.041) | (0.041) | (0.041) | (0.041) | (0.041) | (0.041) | (0.041) | (0.041) | (0.041) | (0.041) |
| Income | -0.008*** | -0.008*** | -0.008*** | -0.008*** | -0.008*** | -0.008*** | -0.008*** | -0.008*** | -0.008*** | -0.008*** | -0.008*** | -0.008*** | -0.008*** |
|  | (0.001) | (0.001) | (0.001) | (0.001) | (0.001) | (0.001) | (0.001) | (0.001) | (0.001) | (0.001) | (0.001) | (0.001) | (0.001) |
| Poverty | 1.466*** | 1.466*** | 1.455*** | 1.460*** | 1.468*** | 1.460*** | 1.465*** | 1.461*** | 1.460*** | 1.461*** | 1.463*** | 1.454*** | 1.460*** |
|  | (0.142) | (0.142) | (0.141) | (0.143) | (0.143) | (0.143) | (0.143) | (0.143) | (0.143) | (0.143) | (0.142) | (0.142) | (0.143) |
| Unemployment | 0.540*** | 0.540*** | 0.560*** | 0.551*** | 0.545*** | 0.550*** | 0.551*** | 0.550*** | 0.545*** | 0.549*** | 0.542*** | 0.562*** | 0.550*** |
|  | (0.205) | (0.204) | (0.203) | (0.206) | (0.205) | (0.205) | (0.206) | (0.206) | (0.205) | (0.207) | (0.205) | (0.203) | (0.206) |
| Uninsured | 0.524*** | 0.523*** | 0.519*** | 0.532*** | 0.526*** | 0.521*** | 0.528*** | 0.527*** | 0.524*** | 0.530*** | 0.525*** | 0.522*** | 0.532*** |
|  | (0.080) | (0.080) | (0.079) | (0.080) | (0.080) | (0.080) | (0.080) | (0.080) | (0.080) | (0.080) | (0.080) | (0.079) | (0.080) |
| Gini | 1.164*** | 1.162*** | 1.139*** | 1.173*** | 1.170*** | 1.172*** | 1.173*** | 1.173*** | 1.173*** | 1.173*** | 1.163*** | 1.143*** | 1.173*** |
|  | (0.126) | (0.126) | (0.125) | (0.127) | (0.127) | (0.127) | (0.128) | (0.128) | (0.127) | (0.128) | (0.126) | (0.126) | (0.127) |
| Elderly | -0.595*** | -0.597*** | -0.605*** | -0.600*** | -0.594*** | -0.602*** | -0.600*** | -0.602*** | -0.604*** | -0.601*** | -0.599*** | -0.608*** | -0.601*** |
|  | (0.058) | (0.058) | (0.057) | (0.058) | (0.058) | (0.058) | (0.058) | (0.058) | (0.058) | (0.058) | (0.058) | (0.057) | (0.058) |
| Nonwhite | 0.210*** | 0.207*** | 0.208*** | 0.209*** | 0.209*** | 0.210*** | 0.209*** | 0.209*** | 0.209*** | 0.209*** | 0.207*** | 0.206*** | 0.209*** |
|  | (0.021) | (0.021) | (0.021) | (0.021) | (0.021) | (0.021) | (0.021) | (0.021) | (0.021) | (0.021) | (0.021) | (0.021) | (0.021) |
| Constant | -1.775*** | -1.772*** | -1.764*** | -1.775*** | -1.776*** | -1.774*** | -1.775*** | -1.774*** | -1.773*** | -1.774*** | -1.772*** | -1.764*** | -1.775*** |
|  | (0.038) | (0.038) | (0.038) | (0.038) | (0.038) | (0.038) | (0.038) | (0.038) | (0.038) | (0.038) | (0.038) | (0.038) | (0.038) |
|  |  |  |  |  |  |  |  |  |  |  |  |  |  |
| Observations | 3,100 | 3,100 | 3,100 | 3,100 | 3,100 | 3,100 | 3,100 | 3,100 | 3,100 | 3,100 | 3,100 | 3,100 | 3,100 |
| Robust standard errors in parentheses; *** p<0.01, ** p<0.05, * p<0.1 | | | |  |  |  |  |  |  |  |  |  |  |

Table 2. OLS regression results – Dependent variable: Poor physical

|  | (1) | (2) | (3) | (4) | (5) | (6) | (7) | (8) | (9) | (10) | (11) | (12) | (13) |
| --- | --- | --- | --- | --- | --- | --- | --- | --- | --- | --- | --- | --- | --- |
|  |  |  |  |  |  |  |  |  |  |  |  |  |  |
| Violation event | 0.025** |  |  |  |  |  |  |  |  |  |  |  |  |
|  | (0.012) |  |  |  |  |  |  |  |  |  |  |  |  |
| Violations |  | 0.002*** |  |  |  |  |  |  |  |  |  |  |  |
|  |  | (0.001) |  |  |  |  |  |  |  |  |  |  |  |
| Pop affected by violations |  |  | 0.002*** |  |  |  |  |  |  |  |  |  |  |
|  |  |  | (0.000) |  |  |  |  |  |  |  |  |  |  |
| Violations duration |  |  |  | 0.000 |  |  |  |  |  |  |  |  |  |
|  |  |  |  | (0.000) |  |  |  |  |  |  |  |  |  |
| T1 violations |  |  |  |  | -0.001 |  |  |  |  |  |  |  |  |
|  |  |  |  |  | (0.003) |  |  |  |  |  |  |  |  |
| Pop affected by T1 violations |  |  |  |  |  | 0.000 |  |  |  |  |  |  |  |
|  |  |  |  |  |  | (0.000) |  |  |  |  |  |  |  |
| T1 violations duration |  |  |  |  |  |  | -0.000 |  |  |  |  |  |  |
|  |  |  |  |  |  |  | (0.000) |  |  |  |  |  |  |
| T1 violations (pathogens) |  |  |  |  |  |  |  | 0.007 |  |  |  |  |  |
|  |  |  |  |  |  |  |  | (0.006) |  |  |  |  |  |
| Pop affected by T1 violations (pathogens) |  |  |  |  |  |  |  |  | 0.000 |  |  |  |  |
|  |  |  |  |  |  |  |  |  | (0.001) |  |  |  |  |
| Pathogens violations duration |  |  |  |  |  |  |  |  |  | 0.000 |  |  |  |
|  |  |  |  |  |  |  |  |  |  | (0.000) |  |  |  |
| T2 violations |  |  |  |  |  |  |  |  |  |  | 0.002*** |  |  |
|  |  |  |  |  |  |  |  |  |  |  | (0.001) |  |  |
| Pop affected by T2 violations |  |  |  |  |  |  |  |  |  |  |  | 0.002*** |  |
|  |  |  |  |  |  |  |  |  |  |  |  | (0.000) |  |
| T2 violations duration |  |  |  |  |  |  |  |  |  |  |  |  | 0.000 |
|  |  |  |  |  |  |  |  |  |  |  |  |  | (0.000) |
| Education | -0.022*** | -0.022*** | -0.022*** | -0.022*** | -0.022*** | -0.022*** | -0.022*** | -0.022*** | -0.022*** | -0.022*** | -0.022*** | -0.022*** | -0.022*** |
|  | (0.001) | (0.001) | (0.001) | (0.001) | (0.001) | (0.001) | (0.001) | (0.001) | (0.001) | (0.001) | (0.001) | (0.001) | (0.001) |
| Income | -0.011*** | -0.011*** | -0.011*** | -0.011*** | -0.011*** | -0.011*** | -0.011*** | -0.011*** | -0.011*** | -0.011*** | -0.011*** | -0.011*** | -0.011*** |
|  | (0.002) | (0.002) | (0.002) | (0.002) | (0.002) | (0.002) | (0.002) | (0.002) | (0.002) | (0.002) | (0.002) | (0.002) | (0.002) |
| Poverty | 0.043*** | 0.043*** | 0.042*** | 0.042*** | 0.042*** | 0.042*** | 0.042*** | 0.042*** | 0.042*** | 0.042*** | 0.042*** | 0.042*** | 0.042*** |
|  | (0.003) | (0.003) | (0.003) | (0.003) | (0.003) | (0.003) | (0.003) | (0.003) | (0.003) | (0.003) | (0.003) | (0.003) | (0.003) |
| Unemployment | 0.036*** | 0.036*** | 0.037*** | 0.036*** | 0.037*** | 0.037*** | 0.037*** | 0.036*** | 0.036*** | 0.036*** | 0.036*** | 0.037*** | 0.036*** |
|  | (0.004) | (0.004) | (0.004) | (0.004) | (0.004) | (0.004) | (0.004) | (0.004) | (0.004) | (0.004) | (0.004) | (0.004) | (0.004) |
| Uninsured | 0.006*** | 0.006*** | 0.006*** | 0.006*** | 0.006*** | 0.006*** | 0.006*** | 0.006*** | 0.006*** | 0.006*** | 0.006*** | 0.006*** | 0.006*** |
|  | (0.002) | (0.002) | (0.002) | (0.002) | (0.002) | (0.002) | (0.002) | (0.002) | (0.002) | (0.002) | (0.002) | (0.002) | (0.002) |
| Gini | 1.735*** | 1.736*** | 1.703*** | 1.752*** | 1.753*** | 1.752*** | 1.753*** | 1.752*** | 1.751*** | 1.751*** | 1.735*** | 1.707*** | 1.751*** |
|  | (0.279) | (0.279) | (0.278) | (0.280) | (0.280) | (0.280) | (0.280) | (0.280) | (0.280) | (0.280) | (0.279) | (0.278) | (0.280) |
| Elderly | -0.013*** | -0.013*** | -0.014*** | -0.014*** | -0.014*** | -0.014*** | -0.014*** | -0.014*** | -0.014*** | -0.014*** | -0.014*** | -0.014*** | -0.014*** |
|  | (0.001) | (0.001) | (0.001) | (0.001) | (0.001) | (0.001) | (0.001) | (0.001) | (0.001) | (0.001) | (0.001) | (0.001) | (0.001) |
| Nonwhite | -0.005*** | -0.005*** | -0.005*** | -0.005*** | -0.005*** | -0.005*** | -0.005*** | -0.005*** | -0.005*** | -0.005*** | -0.005*** | -0.005*** | -0.005*** |
|  | (0.001) | (0.001) | (0.001) | (0.001) | (0.001) | (0.001) | (0.001) | (0.001) | (0.001) | (0.001) | (0.001) | (0.001) | (0.001) |
| Constant | 3.465*** | 3.470*** | 3.481*** | 3.464*** | 3.465*** | 3.465*** | 3.467*** | 3.466*** | 3.466*** | 3.466*** | 3.471*** | 3.482*** | 3.464*** |
|  | (0.088) | (0.088) | (0.088) | (0.088) | (0.088) | (0.088) | (0.088) | (0.088) | (0.088) | (0.088) | (0.088) | (0.088) | (0.088) |
|  |  |  |  |  |  |  |  |  |  |  |  |  |  |
| Observations | 3,100 | 3,100 | 3,100 | 3,100 | 3,100 | 3,100 | 3,100 | 3,100 | 3,100 | 3,100 | 3,100 | 3,100 | 3,100 |
| Adjusted R-squared | 0.729 | 0.729 | 0.730 | 0.729 | 0.729 | 0.729 | 0.729 | 0.729 | 0.729 | 0.729 | 0.729 | 0.730 | 0.729 |
| Robust standard errors in parentheses; *** p<0.01, ** p<0.05, * p<0.1 | | | |  |  |  |  |  |  |  |  |  |  |

Table 3. OLS regression results – Dependent variable: Poor mental

|  | (1) | (2) | (3) | (4) | (5) | (6) | (7) | (8) | (9) | (10) | (11) | (12) | (13) |
| --- | --- | --- | --- | --- | --- | --- | --- | --- | --- | --- | --- | --- | --- |
|  |  |  |  |  |  |  |  |  |  |  |  |  |  |
| Violation event | 0.060*** |  |  |  |  |  |  |  |  |  |  |  |  |
|  | (0.017) |  |  |  |  |  |  |  |  |  |  |  |  |
| Violations |  | 0.004*** |  |  |  |  |  |  |  |  |  |  |  |
|  |  | (0.001) |  |  |  |  |  |  |  |  |  |  |  |
| Pop affected by violations |  |  | 0.003*** |  |  |  |  |  |  |  |  |  |  |
|  |  |  | (0.000) |  |  |  |  |  |  |  |  |  |  |
| Violations duration |  |  |  | 0.000* |  |  |  |  |  |  |  |  |  |
|  |  |  |  | (0.000) |  |  |  |  |  |  |  |  |  |
| T1 violations |  |  |  |  | -0.002 |  |  |  |  |  |  |  |  |
|  |  |  |  |  | (0.005) |  |  |  |  |  |  |  |  |
| Pop affected by T1 violations |  |  |  |  |  | 0.001 |  |  |  |  |  |  |  |
|  |  |  |  |  |  | (0.001) |  |  |  |  |  |  |  |
| T1 violations duration |  |  |  |  |  |  | -0.000 |  |  |  |  |  |  |
|  |  |  |  |  |  |  | (0.000) |  |  |  |  |  |  |
| T1 violations (pathogens) |  |  |  |  |  |  |  | 0.027** |  |  |  |  |  |
|  |  |  |  |  |  |  |  | (0.011) |  |  |  |  |  |
| Pop affected by T1 violations (pathogens) |  |  |  |  |  |  |  |  | 0.001 |  |  |  |  |
|  |  |  |  |  |  |  |  |  | (0.001) |  |  |  |  |
| Pathogens violations duration |  |  |  |  |  |  |  |  |  | 0.000 |  |  |  |
|  |  |  |  |  |  |  |  |  |  | (0.000) |  |  |  |
| T2 violations |  |  |  |  |  |  |  |  |  |  | 0.005*** |  |  |
|  |  |  |  |  |  |  |  |  |  |  | (0.001) |  |  |
| Pop affected by T2 violations |  |  |  |  |  |  |  |  |  |  |  | 0.003*** |  |
|  |  |  |  |  |  |  |  |  |  |  |  | (0.001) |  |
| T2 violations duration |  |  |  |  |  |  |  |  |  |  |  |  | 0.000** |
|  |  |  |  |  |  |  |  |  |  |  |  |  | (0.000) |
| Education | -0.010*** | -0.009*** | -0.009*** | -0.009*** | -0.009*** | -0.009*** | -0.009*** | -0.009*** | -0.009*** | -0.009*** | -0.009*** | -0.009*** | -0.009*** |
|  | (0.001) | (0.001) | (0.001) | (0.001) | (0.002) | (0.002) | (0.002) | (0.001) | (0.002) | (0.002) | (0.001) | (0.001) | (0.001) |
| Income | -0.012*** | -0.012*** | -0.012*** | -0.012*** | -0.012*** | -0.012*** | -0.012*** | -0.012*** | -0.012*** | -0.012*** | -0.012*** | -0.012*** | -0.012*** |
|  | (0.003) | (0.003) | (0.003) | (0.003) | (0.003) | (0.003) | (0.003) | (0.003) | (0.003) | (0.003) | (0.003) | (0.003) | (0.003) |
| Poverty | 0.029*** | 0.029*** | 0.028*** | 0.029*** | 0.029*** | 0.029*** | 0.029*** | 0.029*** | 0.029*** | 0.029*** | 0.029*** | 0.028*** | 0.029*** |
|  | (0.004) | (0.004) | (0.003) | (0.004) | (0.004) | (0.004) | (0.004) | (0.004) | (0.004) | (0.004) | (0.004) | (0.003) | (0.004) |
| Unemployment | 0.056*** | 0.056*** | 0.057*** | 0.057*** | 0.057*** | 0.057*** | 0.057*** | 0.057*** | 0.057*** | 0.057*** | 0.056*** | 0.057*** | 0.057*** |
|  | (0.006) | (0.006) | (0.006) | (0.006) | (0.006) | (0.006) | (0.006) | (0.006) | (0.006) | (0.006) | (0.006) | (0.006) | (0.006) |
| Uninsured | -0.001 | -0.001 | -0.001 | -0.001 | -0.001 | -0.001 | -0.001 | -0.001 | -0.001 | -0.001 | -0.001 | -0.001 | -0.001 |
|  | (0.002) | (0.002) | (0.002) | (0.002) | (0.002) | (0.002) | (0.002) | (0.002) | (0.002) | (0.002) | (0.002) | (0.002) | (0.002) |
| Gini | 2.200*** | 2.206*** | 2.162*** | 2.239*** | 2.242*** | 2.239*** | 2.241*** | 2.241*** | 2.239*** | 2.236*** | 2.203*** | 2.168*** | 2.239*** |
|  | (0.363) | (0.363) | (0.363) | (0.364) | (0.365) | (0.365) | (0.365) | (0.365) | (0.365) | (0.365) | (0.363) | (0.363) | (0.364) |
| Elderly | -0.009*** | -0.009*** | -0.009*** | -0.009*** | -0.009*** | -0.009*** | -0.009*** | -0.009*** | -0.009*** | -0.009*** | -0.009*** | -0.009*** | -0.009*** |
|  | (0.002) | (0.002) | (0.002) | (0.002) | (0.002) | (0.002) | (0.002) | (0.002) | (0.002) | (0.002) | (0.002) | (0.002) | (0.002) |
| Nonwhite | -0.008*** | -0.008*** | -0.008*** | -0.008*** | -0.008*** | -0.008*** | -0.008*** | -0.008*** | -0.008*** | -0.008*** | -0.008*** | -0.008*** | -0.008*** |
|  | (0.001) | (0.001) | (0.001) | (0.001) | (0.001) | (0.001) | (0.001) | (0.001) | (0.001) | (0.001) | (0.001) | (0.001) | (0.001) |
| Constant | 4.426*** | 4.435*** | 4.451*** | 4.419*** | 4.426*** | 4.426*** | 4.427*** | 4.430*** | 4.428*** | 4.428*** | 4.439*** | 4.452*** | 4.422*** |
|  | (0.117) | (0.117) | (0.117) | (0.117) | (0.117) | (0.117) | (0.117) | (0.117) | (0.117) | (0.117) | (0.117) | (0.117) | (0.117) |
|  |  |  |  |  |  |  |  |  |  |  |  |  |  |
| Observations | 3,100 | 3,100 | 3,100 | 3,100 | 3,100 | 3,100 | 3,100 | 3,100 | 3,100 | 3,100 | 3,100 | 3,100 | 3,100 |
| Adjusted R-squared | 0.429 | 0.430 | 0.431 | 0.428 | 0.427 | 0.427 | 0.427 | 0.428 | 0.427 | 0.427 | 0.430 | 0.431 | 0.428 |
| Robust standard errors in parentheses; *** p<0.01, ** p<0.05, * p<0.1 | | | |  |  |  |  |  |  |  |  |  |  |

**Table 4. Updated OLS results used in calculating the marginal diminishing effects of costs associated with health-based violations**

|  | (1) | (2) |
| --- | --- | --- |
| Dependent variable | Poor physical | Poor mental |
|  |  |  |
| Number of violations | 0.00390*** | 0.00959*** |
|  | (0.00132) | (0.00198) |
| Number of violations ^2^ | -0.00003* | -0.00008*** |
|  | (0.00002) | (0.00002) |
| Education | -0.02239*** | -0.00932*** |
|  | (0.00110) | (0.00150) |
| Income | -0.01131*** | -0.01245*** |
|  | (0.00231) | (0.00286) |
| Poverty | 0.04251*** | 0.02880*** |
|  | (0.00290) | (0.00350) |
| Unemployment | 0.03612*** | 0.05608*** |
|  | (0.00434) | (0.00608) |
| Uninsured | 0.00614*** | -0.00159 |
|  | (0.00158) | (0.00208) |
| Gini | 1.72957*** | 2.18802*** |
|  | (0.27845) | (0.36187) |
| Old | -0.01348*** | -0.00864*** |
|  | (0.00141) | (0.00193) |
| Nonwhite | -0.00497*** | -0.00772*** |
|  | (0.00054) | (0.00071) |
| Constant | 3.47342*** | 4.44590*** |
|  | (0.08768) | (0.11678) |
|  |  |  |
| Observations | 3,100 | 3,100 |
| Adjusted R-squared | 0.72917 | 0.43074 |
| Robust standard errors in parentheses | | |
| *** p<0.01, ** p<0.05, * p<0.1 | | |

**Table 5. Diminishing marginal costs of health-based violations (physically unhealthy days)**

| Violations | Total Cost $ | Marginal Cost $  (Δ per violation) | Marginal Cost Decline (%) |
| --- | --- | --- | --- |
| 0 | 0 | – | – |
| 1 | 3,480,403 | 3,480,403 | – |
| 2 | 6,906,846 | 3,426,443 | -1.60% |
| 3 | 10,279,330 | 3,372,484 | -3.10% |
| 4 | 13,597,854 | 3,318,524 | -4.70% |
| 5 | 16,862,418 | 3,264,564 | -6.20% |
| 6 | 20,073,022 | 3,210,604 | -7.80% |
| 7 | 23,229,667 | 3,156,645 | -9.30% |
| 8 | 26,332,352 | 3,102,685 | -10.90% |
| 9 | 29,381,077 | 3,048,725 | -12.40% |
| 10 | 32,375,843 | 2,994,766 | -14.00% |

**Table 6. Diminishing marginal costs of health-based violations (mentally unhealthy days)**

| Violations | Total Cost $ | Marginal Cost $  (Δ per violation) | Marginal Cost Decline (%) |
| --- | --- | --- | --- |
| 0 | 0 | – | – |
| 1 | 4,846,484 | 4,846,484 | – |
| 2 | 9,611,428 | 4,764,944 | -1.70% |
| 3 | 14,294,834 | 4,683,406 | -3.40% |
| 4 | 18,896,700 | 4,601,866 | -5.00% |
| 5 | 23,417,027 | 4,520,327 | -6.70% |
| 6 | 27,855,815 | 4,438,788 | -8.40% |
| 7 | 32,213,064 | 4,357,249 | -10.10% |
| 8 | 36,488,774 | 4,275,710 | -11.80% |
| 9 | 40,682,944 | 4,194,170 | -13.50% |
| 10 | 44,795,575 | 4,112,631 | -15.10% |

**Note that:**

1. **Marginal Cost** (Δ per violation) is calculated as:

$$(\beta_{1}+2\beta_{2}\times Number of violations)\times AC\times Population$$

Where:

$\beta_{1}$ is the coefficient for Number of violations

$\beta_{2}$ is the coefficient for Number of violations ^2^

$AC$ is the annual cost per unhealthy day obtained from McNamara et al. (2024). For physically unhealthy days, $AC=720 (60\times12)$ and for mentally unhealthy days, $AC=408 \left( 34\times12 \right)$

$Population$ is the county’s average population in the US, which is 104,089

1. **Total Costs** are the sum of marginal costs up to that violation.
